# Live genome imaging by CRISPR engineering: progress and problems

**DOI:** 10.1038/s12276-025-01498-x

**Published:** 2025-07-31

**Authors:** Eui-Jin Park, Hajin Kim

**Affiliations:** Department of Biomedical Engineering, Ulsan National Institute of Science and Technology, Ulsan, Republic of Korea

**Keywords:** Chromatin structure, Super-resolution microscopy

## Abstract

CRISPR–Cas-based genome imaging opened a new era of genome visualization in living cells. While genomic loci with repetitive sequences, such as centromeres and telomeres, can be reliably imaged, applying the technique to nonrepetitive genomic loci has remained challenging. Recent advancements in the design of CRISPR RNAs and Cas proteins, the development of novel fluorophores and the combination of CRISPR–Cas with other molecular machinery amplified target-specific signals and suppressed background signals, revolutionizing this unique genome imaging technique and enabling the tracking of genomic loci with a small number of CRISPR–Cas complexes, down to a single complex. Here we review the latest advancements in CRISPR–Cas-based genome imaging techniques and their application to imaging nonrepetitive genomic loci. The challenges that these techniques are currently facing are the cellular toxicity and genomic instability induced by the expression of CRISPR–Cas and its interference with DNA metabolism, which impacts DNA replication and genome maintenance. Recently reported adverse effects of CRISPR–Cas-based genome labeling are discussed here, along with perspectives on how to overcome the problem.

## Enhancing genome visualization with CRISPR technology

Understanding how our DNA is organized in three dimensions and changes over time is important for studying how genes work. This Review highlights the use of CRISPR–Cas9, a tool originally for gene editing, now adapted for live-cell imaging of DNA. Researchers use a catalytically dead Cas9 and, by attaching fluorescent proteins to the complex, label specific DNA regions in live cells. This technique faces challenges in imaging nonrepetitive DNA regions, which requires transfection with multiple guide RNAs. New techniques amplify signal and reduce background, making it possible to visualize a target even with a single CRISPR complex. Emerging evidence suggests that CRISPR–Cas9 for imaging interferes with genomic processes. The authors suggest that future research should focus on refining these methods to minimize side effects and improve delivery systems for CRISPR components.

This summary was initially drafted using artificial intelligence, then revised and fact-checked by the author.

## Introduction

Visualizing the three-dimensional (3D) organization of genome and its dynamic changes is crucial to understanding the regulation of genomic processes^1–4^. Hi-C techniques provide genome-wide information on the hierarchical organization of chromosomal domains with kilobase resolution, and fluorescence in situ hybridization (FISH) techniques show the spatial arrangement of genomic regions^5–7^. These techniques, however, are applicable mostly to fixed cells and do not show how genome organization changes over time, despite several recent works that applied FISH techniques to living cells, but under harsh conditions questioning cell viability^8–10^. Reliable live-cell chromatin imaging is essential for capturing the spatiotemporal behavior of genome and providing insights into the dynamic interaction of genome with nuclear components. These insights elucidate the mechanisms of chromatin compartmentalization^11^, cell differentiation^12,13^, development^14^ and diseases^15,16^, where genome organization plays important roles.

CRISPR–Cas9, originally adopted for genome editing, has expanded its scope of applications to many areas, including chromatin imaging^17,18^. The development of a nuclease-deactivated variant of Cas9 (dCas9), which retains the ability to recognize and bind target DNA, was crucial^19^. By fusing dCas9 with fluorescent proteins such as eGFP or mCherry, CRISPR has been harnessed for live genome imaging with high target specificity, marking the beginning of a new era in chromatin imaging^20–26^.

Despite these advances, imaging an arbitrary genomic region is still challenging. CRISPR–Cas-based genome imaging often targets genomic regions with repetitive sequences such as centromeres, alpha satellites and telomeres, which are easier to visualize than nonrepetitive regions owing to the abundance in CRISPR targets with a single kind of guide RNA (gRNA)^27,28^. Imaging genomic loci with nonrepetitive sequences requires the incorporation of a large number of gRNAs and optimization of the CRISPR design and imaging conditions to maximize signal-to-noise ratios (SNRs)^20,26^. Varying conditions and microscopy techniques make it difficult to compare the reported methods and find applicable solutions. This Review aims to provide a comprehensive overview of the current CRISPR–Cas-based genome imaging methods, their performances in imaging nonrepetitive genomic loci and the side effects of CRISPR–Cas-based genome imaging on cell viability and genomic processes, to aid future research in adopting these methods.

## Recent developments in CRISPR–Cas-based genome imaging

### Overview of CRISPR–Cas-based genome imaging

CRISPR–Cas-based imaging leverages the unique properties of Cas proteins to bind a double-stranded DNA with a sequence defined by the CRISPR RNA (crRNA), which is typically linked to another trans-encoded RNA (tracrRNA) to make a single guide RNA (sgRNA), which forms a complex with a Cas protein ^20^. Cas9 protein is mutated to be nuclease-deficient (dCas9) so that it binds to the target DNA without cleaving the DNA, and fluorescent proteins are fused to dCas9 or engineered to bind the gRNA scaffold^20,21,23–26^. Orthologous CRISPR systems from multiple bacterial species were exploited to image multiple targets simultaneously^21,22^. dCas9 of each species recognizes its matching sgRNA and a unique protospacer adjacent motif sequence on the DNA.

Initial designs using dCas9 fused with a fluorophore experienced high background levels and aggregation. This was mitigated by avoiding the placement of labels directly on dCas9 and by using split fluorophores. Split fragments of the fluorescent protein GFP formed full fluorophores on assembled CRISPR complexes and greatly reduced nonspecific background signals^29^. Multilocus imaging was also achieved by incorporating various RNA aptamers such as MS2 and PP7 motifs into sgRNA scaffolds and using fluorescent proteins linked to MCP and PCP, which bind these motifs^22,25^. sgRNAs with protein-binding scaffolds were also used for signal amplification, as repeated protein-binding motifs allowed the recruitment of multiple fluorescent proteins and amplified signals^23,24,26,30–32^. RNA-binding proteins such as MCP, PP7, Com and lambdaN were utilized for orthogonal labeling of sgRNA^23,31,33–35^. Another method for signal amplification is the SunTag system, which uses a GCN4 peptide array to recruit scFv fused to superfolder GFP (sfGFP)^36^. In this work, dCas9 fused to 24-repeat SunTag enhanced the signal brightness 19-fold compared with dCas9–EGFP in HEK293 cells. The development of various CRISPR–Cas-based imaging systems has led to significant breakthroughs in understanding chromatin dynamics and nuclear architecture. Long-term live tracking of genomic loci has enabled precise analysis of their diffusion behaviors, which are influenced by factors such as location in the nucleus, cell cycle, metabolic state and DNA damage^20,35,37–39^.

### Novel genome imaging techniques based on CRISPR–Cas

Despite the advancements in CRISPR–Cas-based genome imaging, most applications of the technique focused on targets with repetitive sequences, such as centromeres, satellites and telomeres^25,30,40,41^. Genomic regions containing repetitive sequences are easier to target owing to the high abundance of target sequences, allowing efficient imaging with a minimal set of sgRNAs. Targeting nonrepetitive regions is challenging as the simultaneous transfection with multiple plasmids is difficult^20,26^. Transfecting the cell with a plasmid containing multiple sgRNAs provides a plausible solution to this, but the binding of CRISPR–Cas complexes to a group of distinct targets is not as efficient as that for repetitive targets^42^.

Recent developments of CRISPR–Cas systems for improving the labeling efficiency and coverage are summarized in Table 1. CRISPR-Sirius enhances signal strength and stability by inserting repeated RNA aptamer sequences such as MS2 and PP7 into the tetraloop of sgRNA^43^. The design of the insert was optimized by randomizing the linker between the RNA aptamers, making synonymous mutations to avoid long repeats in the sequence and minimizing undesired RNA secondary structures. This design improves the stability of sgRNA and allows higher-resolution imaging of genomic loci. Splitting sfGFP into three fragments and integrating these with the SunTag system and extended sgRNA scaffolds greatly reduced background noise and facilitated fluorescence recovery by the frequent disassembly–reassembly of the sfGFP fragments, thereby allowing reliable long-term imaging^35^. CRISPR/Casilio uses an sgRNA containing multiple Pumilio/FBF (PUF) binding sites and PUF fused with fluorescent proteins such as Clover, iRFP670 and mRuby2 to amplify the signals, enabling high-resolution, multiplexed imaging of chromatin interactions^44^.

**Table 1:** Novel CRISPR–Cas-based genome imaging techniques.

Live/fixed cells	Name of technology	Type of Cas	Composition of CRISPR–Cas complex	Type of target region	Features	Reference
Live cells	CRISPR-Sirius	dCas9	dCas9, aptamers inserted into the tetraloop of sgRNA	repetitive	Improved gRNA stability by modifying RNA scaffolds, better signal amplification than conventional methods	Ma et al.^43^
Live cells	Tripartite sfGFP	dCas9	GFP1-9, scFv-GFP10, MCP-mCherry-GFP11, dCas9-24X GCN4, sgRNA 12XMBS	Repetitive/nonrepetitive	Enhanced SNR by decreasing background level and amplifying signals using tripartite GFP and SunTag	Chaudhary et al.^35^
Fixed cells	Genome oligopaint via local denaturation (GOLD) FISH	Cas9 nickase	Cas9 nickase, gRNA, superhelicase, Cy5-FISH probe	Repetitive/nonrepetitive	Increased specificity and amplified signal by local DNA denaturation for FISH probe access	Wang et al.^45^
Live cells	CRISPR FISHer	dCas9	dCas9, sgRNA-multi PP7, Foldon-GFP-PCP	Repetitive/nonrepetitive	Signal amplification mediated by phase separation in live cells	Lyu et al.^46^
Live cells	CRISPR/Casilio-based imaging	dCas9	dCas9, gRNA-PBS, PUF-fluorescent protein (FP)	Nonrepetitive	Amplifiable and multiplexable imaging by integrating PUF binding sequences in sgRNA	Clow et al.^44^
Live cells	CRISPR-SunTag with LEXY	dCas9	dCas9-SunTag, scFv-sfGFP-LEXY	Repetitive	Improved SNR by optically controlled export of untargeted fluorescent proteins to the cytoplasm	Hou et al.^47^
Fixed cells	CasSABER	dCas9	dCas9/sgRNA, primer-exchange reaction (PER) probes, imager-fluorescent tag	Nonrepetitive	Signal amplification by branching primer-exchange reaction probes	Li et al.^48^
Live cells	Fluorogenic CRISPR (fCRISPR)	dCas9	dCas9, sgRNA with Pepper aptamers, FP–tDeg	repetitive	Enhanced SNR by using degradable fluorogenic proteins to be stabilized by target binding	Zhang et al.^49^
Live cells	CRISPR/Pepper-tDeg	dCas9	dCas9, sgRNA with the degron binding Pepper aptamers, split FP–tDeg	Repetitive/nonrepetitive	Enhanced SNR by combining tDeg-based fluorogenic CRISPR and MS2-MCP system	Chen et al.^50^
Live cells	CRISPRdelight	dCas12a	dCas12a, engineered CRISPR array for multiplexed imaging	Nonrepetitive	Signal amplification and multiplexing by processing CRISPR arrays with dCas12a	Yang et al.^57^

Unconventional ways to amplify target CRISPR signals have been reported. GOLD FISH eliminates the need for multiple CRISPR complexes targeting a single genomic region by using a Cas9 nickase and a superhelicase to unwind target DNA regions and labeling them with conventional FISH probes, which requires the fixation of cells^45^. This method allows precise labeling of both repetitive and nonrepetitive sequences with enhanced specificity and expands the targeting capability of CRISPR imaging. Another method, CRISPR FISHer, exploits phase separation to mediate the amplification of target-specific fluorescence signals, using an engineered sgRNA scaffold coupled with a trimeric foldon–GFP fusion protein^46^. It showed a 246-fold improvement in SNR compared with the conventional dCas9–eGFP design, facilitating real-time tracking of chromosomal events, such as chromatin dissociation and intra- and interchromosomal rejoining induced by DNA double-strand breaks. An optogenetically controlled system, which integrates a light-inducible nuclear export tag (LEXY) with a CRISPR-SunTag system, showed a ~2–2.5 fold improvement of SNR by selective removal of untargeted fluorescent proteins from the nucleus^47^. A signal amplification method based on primer-exchange reaction, CRISPR–Cas-mediated signal amplification by exchange reaction (CasSABER), enhances fluorescent signals by multiple rounds of branching hybridization^48^.

Recently developed CRISPR labeling methods, fCRISPR and CRISPR/Pepper-tDeg, used a novel fluorogenic protein^49,50^. Fusing a fluorescent protein with a degron domain derived from Tat peptide (tDeg), which is protected from degradation only when binding an RNA aptamer, Pepper, untargeted fluorophores were eliminated and a much higher SNR was achieved. The CRISPR/Pepper-tDeg technique also fused tDeg with a tandem repeat of a split GFP fragment, GFP_11_, to amplify signals by assembling this with separately expressed GFP_1–10_ fragments^50^. Cas12a has also been exploited for genome imaging^51–54^. Unlike dCas9, dCas12a can process pre-crRNAs into multiple mature crRNAs, providing a solution to transfect cells with multiple sgRNAs^55,56^. The CRISPRdelight technique used a CRISPR–dCas12a array for efficient multiplexed imaging of genomic loci^57^.

### Imaging nonrepetitive genomic loci

Repetitive genomic regions offer an advantage in CRISPR–Cas-based imaging due to the redundancy of targets, but many important biological problems involve the reorganization of nonrepetitive genomic regions. It is challenging to image nonrepetitive genomic loci, because it is required to insert many kinds of sgRNA and, even when they are successfully expressed, each sgRNA needs to find a single binding target. In addition, off-target binding needs to be addressed for each sgRNA.

Recently developed techniques have advanced CRISPR–Cas-based imaging by eliminating background signals, amplifying target-specific signals and improving the targeting accuracy of CRISPR–Cas. Table 2 lists the studies that report successful imaging of nonrepetitive genomic loci using these techniques. Several studies reported imaging a genomic locus with a single CRISPR–Cas complex bound to it^44,46,48,50^. Novel designs of CRISPR systems in these studies all achieved the visualization of nonrepetitive genomic loci using a single sgRNA, but through distinct amplification strategies: Casilio uses Pumilio-mediated recruitment of multiple fluorescent proteins, FISHer exploits protein phase separation, CasSABER leverages iterative primer exchange reaction, and Pepper-tDeg relies on background suppression by target-dependent activation of degron and split-GFP. While Casilio and FISHer provide relatively straightforward designs for signal amplification, CasSABER offers high programmability at the cost of complex probe design and the limited application to fixed cells, and Pepper-tDeg offers high SNR without assembling large number of proteins by degrading fluorescent proteins in the background. As these techniques rely on a single CRISPR complex correctly binding the target, they may face challenges from off-target binding of CRISPR and need finely tuned expression control. Given the varying efficiency of CRISPR editing on different genomic loci, the performance of these methods may also vary depending on the target. The applicability and reliability of these methods for varying target sites need to be addressed in future studies.

**Table 2:** Imaging low-repeat and nonrepetitive genomic loci using CRISPR–Cas.

Labeling strategy	Fluorescent tag	Target site	(Low-repeat targets) Number of gRNAs	(Nonrepetitive targets) Number of gRNAs used/target size	Cell line	Microscope	Reference
MPC, PCP motif	MCP–EGFP, PCP–mCherry	Low-repeat (*Igh*, *Akap6* locus)	Igh: 5–18 locations for each 13 sgRNA, Akap6: 87 locations for a sgRNA	-	Mouse 3T3 fibroblast cells	Wide-field microscope, 3D *z* stack, 3D deconvolution	Fu et al.^30^
MCP, PCP motif, sgRNA 2.0 16x-MS2	MCP–YFP, MCP–mCherry	Low-repeat (MUC4, locus ~#1–4), nonrepetitive (the first intron of the MUC4)	MUC4: 84 repeats for a sgRNA, locus ~#1–4: 8, 15, 21 and 33 repeats, respectively	Intron of MUC4: ~4–30 sgRNAs across 5 kb	HeLa, U2OS and RPE1 cells	Confocal microscope, lattice light-sheet microscope, 3D *z* stack	Qin et al.^26^
MCP, PCP motif, sgRNA-Sirius-8xMS2	MCP–Halo, PCP–GFP, RNA aptamers at tetraloop of the sgRNA scaffold	Low-repeat (C19-1, C19-2, intron 10 in FBN3 and 26 loci in Ch19)	C19-1 (36 copies), C19-2 (45 copies), 22 (for FBN3), 26 loci (≥20 copies)	-	U2OS cells	Leica DM IRB microscope with EMCCD camera and 100× oil lens	Ma et al.^43^
SHACKTeR (short homology and CRISPR–Cas9-mediated knock-in of a TetO repeat)	TetR–EGFP	Nonrepetitive (10 different loci including HSP70 locus), inserted short repeat	48-mer and 96-mer TetO repeats (~3–4.6 kb for TetO repeat integration)	-	HCT116 cells, HEK293T	Wide-field microscope, deconvolution, structured illumination	Tasan et al.^82^
CRISPR/dual-FRET MB (dual molecular beacons for FRET)	Donor MB-Atto550, acceptor MB-Atto647N	Nonrepetitive (MUC4 gene, MUC1 gene, intergenic region)	-	3 unique sgRNAs for each loci/~220–770 bp depending on the locus	HeLa, U2OS cells	Wide-field microscope, 3D *z* stack, deconvolution	Mao et al.^28^
SunTag tripartite sfGFP	GFP1-9, scFv–GFP10, MCP–mCherry–GFP11	Low-repeat (X-114 locus)	13 copies	–	AD-293 cell	Confocal microscope, 63× 1.4 NA lens, 3D *z* stack	Chaudhary et al.^35^
CRISPR FISHer (use Foldon trimerization, sgRNA-8xPP7)	Foldon–GFP–PCP	Nonrepetitive (PPP1R2 gene)	–	sgRNA, single binding site	U2OS, HeLa, HepG2	Nikon Eclipse Ti-E with Andor Sona 4BV6U Camera, Plan APO λ 100× / 1.45 oil objective, 3D *z* stack	Lyu et al.^46^
CRISPR/Casilio (use 15× PUF domain and PUF binding site)	PUF-Clover/iRFP670/mRuby2	Nonrepetitive (MUC4 gene, MASP1–BCL6 loop, IER5L promoter–super enhancer loop)	–	sgRNA, single binding site	U2OS, ARPE-19, HCT116/RAD21-mAID, HAP1	Confocal microscope, 3D *z* stack, 3D drift correction, deconvolution	Clow et al.^44^
CasSABER (use primer exchange reaction probe)	Cy3/Cy5/AF488 - imager	Low-repeat (MUC4 intron, HTT gene), nonrepetitive (MUC4 gene)	MUC4 intron (90 copies), HTT gene (17 copies)	1, 3 and 6 gRNAs	MCF-7, HeLa	Confocal microscope, 3D z stacks	Li et al.^48^
CRISPRdelight (use dCas12a and engineered CRISPR array)	dCas12a–EGFP/StayGold	Nonrepetitive (CCAT1 locus, S100A10, EFNA1, GPBP1, H3C1, MYC, NFIL3, HSPH1)	–	Arrays with 12, 24, 36 and 48 crRNAs	HeLa, U2OS, HCT116	Wide-field microscope, deconvolution	Yang et al.^57^
fCRISPR (use degron binding pepper aptamers)	FP–tDeg	Low-repeat (MUC4 intron 1, Chromosomes 3, 9, 13 and 19 regions)	MUC4 intron 1 (90 copies), Chr19 (30 copies), Chr3 (25 copies), Chr9 (17 copies), Chr13 (14 copies)	-	HEK293T, HeLa, Huh7, LO2, U2OS	Confocal microscope	Zhang et al.^49^
CRISPR/Pepper-tDeg (use degron-binding pepper aptamers at sgRNA)	Split GFP–tDeg	Low-repeat (IDR1, IDR3, FBN3), nonrepetitive (MUC4, IL-1B)	IDR1 (61 copies), IDR3 (45 copies), FBN3 (22 copies)	sgRNA, single binding site	HEK293T	Confocal microscope	Chen et al.^50^

*FRET* Förster resonance energy transfer.

## Adverse effects of CRISPR–Cas-based genome labeling

Increasing evidence suggests that dCas9 binding causes unintended effects on cellular processes. Although dCas9 does not directly cleave the target DNA, it can modulate the accessibility of the DNA to other proteins and interfere with genomic processes such as replication, repair and transcription. Recent reports on the effect of CRISPR–dCas9 binding are summarized in Table 3 and discussed below.

**Table 3:** Adverse effects of dCas9 expression and target binding.

Keyword	Features	Organism/system	Reference
DNA replication	dCas9 binding blocks DNA replication proteins	In vitro (viral, bacterial, eukaryotic)	Whinn et al.^58^
	dCas9 binding destabilizes targeted array and showed copy number variation	Yeast cells	Doi et al.^59^
	dCas9 binding delays replication timing and sister chromatin resolution	Mammalian cells	Xiong et al.^60^
R-loop formation	dCas9 induces mutations due to dCas9-induced R-loop	Yeast cells	Laughery et al.^64^
	dCas9 induced R-loop inhibits the initiation of BER on both strands of the DNA	In vitro	Antony et al.^65^
Chromatin accessibility	dCas9 binding opens chromatin inducing accessibility	Mouse embryonic stem cells (mESC)	Barkal et al.^66^
dCas9 expression	High-level expression of dCas9 induces abnormal cell morphology	*E. coli*	Cho et al.^67^
	dCas9 induces fitness defects depending on dCas9 concentration	*E. coli*	Cui et al.^68^
	dCas9 causes strong growth inhibition in the absence of sgRNA	*C. trachomatis*	Wurihan et al.^69^
Cell cycle	dCas9 causes TP53-dependent cell cycle arrest	Human cells	Geisinger et al.^70^.

### Replication blockage and genomic instability

Several studies reported that dCas9 binding on DNA hinders DNA replication across different biological systems. In vitro experiments confirmed that the CRISPR–dCas9 complex can obstruct the progression of DNA replication complex from viruses, bacteria and eukaryotic cells^58^. Remarkably, at high concentrations, dCas9 alone was shown to inhibit the assembly of replisomes. In *Saccharomyces cerevisiae*, dCas9 binding interferes with DNA replication and generates structural variation^59^. dCas9-bound CUP1 locus, which is a tandem repeat array, showed copy number variation. dCas9 was also shown to impede replication fork progression and accumulate replication intermediates in cultured cells, as revealed by neutral–neutral 2D gel electrophoresis and Southern blot hybridization^59^.

Another study reported a reduced formation of doublet foci, which indicate active DNA replication, during early- and mid-S phases in CRISPR–Cas-expressing cells^60^. The overall duration of DNA replication, from the onset of S phase to its completion, was extended upon CRISPR–Cas-based labeling, compared with TetR-based labeling. Even LacI- or TetR-based labeling can induce replication fork stalling and recruit DNA damage response (DDR) proteins, such as γH2AX, TOPBP1 and 53BP1, indicating the presence of double-strand breaks and subsequent activation of the DDR^61^. CRISPR–Cas-based labeling presumably causes stronger blockage of the replication fork and triggers stronger DDR and genomic instability at the targeted locus.

### R-loop formation

R-loops are three-stranded nucleic acid structures consisting of an RNA–DNA hybrid and a displaced single-stranded DNA, and they have emerged as a key factor in genomic instability^62,63^. CRISPR–dCas9 binding induces the formation of ~20-base-long R-loops. CRISPR–Cas-induced R-loops promote spontaneous cytosine deamination at the exposed single-stranded DNA, leading to mutations^64^. They have also been shown to inhibit base excision repair in vitro, especially uracil lesions^65^. The activity of uracil-DNA glycosylase, a key enzyme in the BER pathway that cleaves uracil-containing DNA, was reduced by 2.6-fold in the presence of CRISPR–dCas9. This suggests that CRISPR–Cas-induced R-loops not only create opportunities for the formation of mutagenic lesions, but also hinder the repair machinery for these lesions, promoting genomic instability.

### Chromatin accessibility and gene expression level

dCas9 binding also alters chromatin accessibility. It was reported that dCas9 binding induces chromatin opening and facilitates the binding of DNase and retinoic acid receptors, thus enhancing the ability of retinoic acid to induce GFP expression^66^. However, the effects and extent of dCas9 binding on gene expression remain contentious. A different study reported that dCas9 binding does not affect the subnuclear location of the labeled loci or the expression of genes at adjacent regions^58^. These observations suggest that the influence of dCas9 on gene expression may depend on the genomic region owing to the varying genomic context and response of regulatory elements.

### Cellular toxicity

The expression level of dCas9 has been shown to correlate with the impact on cellular physiology in various organisms. In *Escherichia coli*, overexpression of dCas9, even without gRNA, causes abnormal cell morphology and a notable reduction in growth rate to approximately 50% of that of wild-type cells^67^. RNA sequencing analysis revealed that 574 genes were differentially transcribed under high dCas9 expression, with particularly large impact on cell division and membrane-associated proteins. In addition, gRNA-sequence-dependent blocking of gene transcription in *E. coli* is observed. This effect produces fitness defects or even kills *E. coli*, depending on the concentration of dCas9^68^. In *Chlamydia trachomatis*, high-level expression of dCas9 from *Streptococcus pyogenes* caused pronounced growth inhibition, in the absence of gRNA^69^. dCas9 from *Staphylococcus aureus* also exhibited strong toxicity when expressed with a nontargeting gRNA scaffold. dCas9 binding can also arrest the cell cycle in a *TP53*-dependent manner^70^. Imaging applications typically require higher dCas9 concentrations compared with gene editing, increasing the risk of unintended cellular stress or toxicity. These findings suggest that dCas9 expression should be carefully regulated, especially when using it as an imaging tool to study chromatin dynamics.

## Conclusions and perspectives

**Fig. 1:**
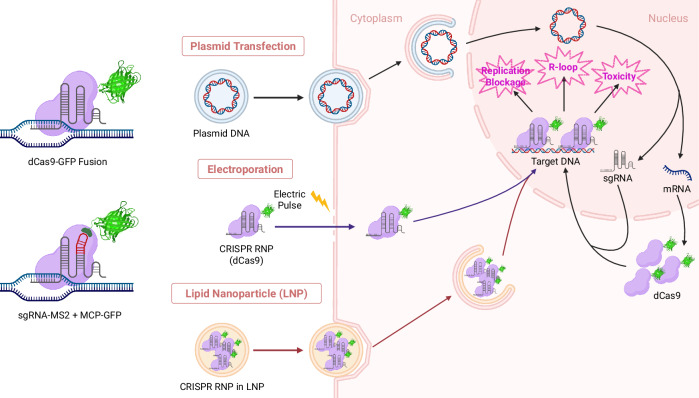
Schematic showing the delivery dCas9 and gRNA delivery into cell nucleus for chromatin imaging. Created with BioRender.com. Three dCas9/gRNA delivery methods are illustrated. (1) Plasmid transfection. Plasmid DNA is delivered to the cell cytoplasm via endocytosis. Upon entry into the nucleus, the plasmid is transcribed to produce sgRNAs and mRNAs to be translated to produce dCas9 proteins in the cytoplasm, which then translocate back to the nucleus using their nuclear localization signal and form dCas9–sgRNA complexes to target genomic loci. (2) Electroporation. Preassembled dCas9–sgRNA RNP complexes are delivered into the cell through membrane pores transiently formed by an electric pulse. (3) Lipid nanoparticle. Preassembled dCas9–sgRNA RNP complexes are encapsulated in lipid nanoparticles and delivered by endocytosis.

CRISPR–Cas-based chromatin imaging techniques have greatly advanced, expanding our understanding of chromatin dynamics during cellular processes. Although further refinements are required for their application to diverse genomic loci and tissue-level studies, recent technical developments have brought us closer to the labeling of nonrepetitive genomic loci with a single sgRNA. One big challenge the techniques are facing is the delivery of CRISPR complexes. High-level expression of dCas9 is essential for effective genome labeling, but excessive expression of dCas9 is detrimental to cell health. Thus, delivering a controlled amount of dCas9 to the nucleus is essential for reliable imaging of genomic loci. Also, artificial organic fluorophores offer many advantages over fluorescent proteins, if they can be delivered to target sites. Currently, three types of approaches are primarily used to deliver CRISPR–Cas complex to the cell (Fig. 1). Plasmid transfection is the most commonly used technique for genome imaging^30,38,40,60,71^. Inducible expression systems have been used to mitigate the overexpression problem^68,69^.

More precise control of dCas9 and sgRNA levels can be achieved by delivering preassembled ribonucleoprotein (RNP) complexes of CRISPR–Cas. RNP delivery into the nucleus is performed primarily by two methods. One is electroporation or nucleofection, which creates temporary pores on the membrane using electric pulses. The pores enable RNPs to enter the cytoplasm and eventually the nucleus^72–74^. However, this technique requires a recovery period to diminish the shock and stress that the electric pulses caused, and the cell survival is generally inefficient^72,74^. Alternatively, lipid nanoparticles are used to encapsulate and deliver CRISPR RNP complexes via endocytosis^75–77^. The technique uses stable, well-characterized lipid nanoparticle formulations. This method is widely used in CRISPR–Cas-based gene editing, which generally requires lower levels of Cas proteins than chromatin labeling does. Delivering sufficient quantities of Cas proteins for chromatin imaging without compromising cellular functions still remains challenging. Advances in RNP delivery methods are anticipated to revolutionize CRISPR–Cas-based genome imaging. One promising approach uses cryo-shocked cells in combination with lipid nanoparticles for CRISPR–Cas delivery^78^. The study demonstrated successful dCas9 plasmid delivery to mouse lung tissue using cryo-shocked tumor cells, maintaining the structural integrity of cells while avoiding pathogenicity. Addressing these issues will be instrumental not only for the field of CRISPR–Cas-based imaging, but also for epigenomic gene regulation and prime editing using dCas9^79–81^. With ongoing progress, CRISPR–Cas-based genome imaging holds promise for broadening our understanding of genome dynamics.
